# The Role of TLR4 in Lung Epithelial Cell Injury Caused by Influenza Virus Combined with *Staphylococcus aureus*

**DOI:** 10.3390/microorganisms13061201

**Published:** 2025-05-24

**Authors:** Bei Chen, Chunjing Chen, Fangguo Lu, Xiaoqi Wang, Xianggang Zhang, Zhibin Wang, Huihui Liu

**Affiliations:** 1 Medical School, Hunan University of Chinese Medicine, Changsha 410208, China; 20223769@stu.hnucm.edu.cn (B.C.); 004787@hnucm.edu.cn (C.C.); liuhuihui916@163.com (H.L.); 2 School of Integrated Chinese and Western Medicine, Hunan University of Chinese Medicine, Changsha 410208, China; 20232123@stu.hnucm.edu.cn (X.W.); 20222126@stu.hnucm.edu.cn (X.Z.); 15680801830@163.com (Z.W.)

**Keywords:** influenza A virus, *Staphylococcus aureus*, secondary infection, inflammatory factor, cell trauma

## Abstract

Influenza A virus (IAV) is a major cause of respiratory illness in humans and animals. Secondary bacterial infections, especially those caused by *Staphylococcus aureus* (SA), significantly increase influenza-related morbidity and mortality. However, the mechanisms underlying these co-infections remain unclear. In this study, we examined how IAV infection influences SA-induced inflammation in lung epithelial cells. Our study was conducted based on in vitro experiments. First, we infected MLE-12 cells with IAV, confirming viral replication and the resulting cell damage. SA was then introduced 24 h or 36 h post-infection, and the cellular responses were measured. We assessed cell viability, cell-free DNA, Citrullinated histone H3, and the mRNA expression of TLR4 and proinflammatory cytokines. Our results showed that IAV+SA stimulation significantly increased upregulated TLR4 expression and inflammatory damage. To further explore TLR4’s role, we used the inhibitor TAK-242 and a TLR4 siRNA knockdown. Both approaches reduced the inflammatory response triggered by IAV and SA stimulation. These findings suggest that TLR4 is a key mediator in the enhanced inflammation observed during IAV and SA co-infection, offering a potential target for therapeutic intervention.

## 1. Introduction

There are three main types of influenza viruses: A, B, and C. Among them, influenza A virus (IAV) is a major respiratory pathogen, causing 3–5 million severe clinical infections and 250,000–500,000 deaths each year. It is a major cause of global disease and death and poses a serious threat to human health [1,2]. Epidemiological data on influenza indicate that the most common complication is secondary bacterial infection, which is one of the leading causes of death among patients with influenza [3,4]. Among the bacteria causing secondary infections, *Streptococcus pneumoniae*, *Staphylococcus aureus* (SA), and *Haemophilus influenzae* are relatively common [5,6]. Among these, SA is considered the most common pathogenic bacterium [7]. SA is a Gram-positive coccus and a primary pathogen in human bacterial infections [8]. Pulmonary invasion by SA is a critical trigger for acute lung injury (ALI) and acute respiratory distress syndrome (ARDS). SA pneumonia progresses rapidly, and if it is not promptly diagnosed and treated, it can lead to infectious death [9,10].

IAV enhances host susceptibility to bacterial infections through multiple mechanisms that promote bacterial adhesion and colonization of the respiratory tract. Lung epithelial cells are the primary target cells of IAV. IAV binds to sialic acid receptors on the host cell surface through hemagglutinin protein, triggering endocytosis. After being endocytosed into the cell, IAV cleaves sialic acid on the cell surface under the action of neuraminidase, causing changes in the surface protein receptors of infected cells, thereby releasing genetic material to initiate viral replication [11]. This process can directly cause epithelial damage and apoptosis, exposing bacterial binding sites and facilitating bacterial invasion into lung tissues [12,13]. Additionally, damaged epithelial cells secrete increased amounts of adhesion factors, which promote bacterial adhesion, colonization, and invasion [14,15], further increasing the host’s susceptibility to bacterial pneumonia [16].

As the organism’s primary protective barrier, the innate immune mechanism rapidly initiates defense responses against pathogenic intrusions at the initial stage of infection. Toll-like receptors (TLRs), a type of pattern recognition receptor (PRR), play a critical role in detecting pathogenic microorganisms and initiating innate immune responses [17]. Toll-like receptor 4 (TLR4), a transmembrane protein expressed by both immune and non-immune cells, is closely associated with cell differentiation, proliferation, apoptosis, and proinflammatory responses. TLR4 has been confirmed as a key regulatory factor in adipocyte differentiation [18]. LPS can mediate cell proliferation through TLR4/MyD88 [19]. Overexpression of TLR4 also promotes apoptotic responses, and furthermore, it is associated with inflammatory reactions [20]. Studies have reported that activation of TLR4 plays a significant role in IAV infection [21]. NF-κB, a downstream effector of TLR4, regulates the expression of multiple genes involved in inflammatory responses [22]. IAV can rapidly induce acute lung injury via the TLR4/NF-κB signaling pathway, while the upregulated expression of TLR4 may further trigger NF-κB activation [23]. TLR4 is traditionally recognized as a receptor for lipopolysaccharide (LPS) in Gram-negative bacteria; however, emerging evidence suggests that its expression is associated with Gram-positive bacterial infections [24].

The pathogenic mechanisms underlying influenza virus and bacterial co-infection remain unclear, complicating the development of control measures and therapeutic interventions. Based on the immunoregulatory mechanisms of TLR4, we hypothesized that co-stimulation with IAV and SA activates TLR4, thereby promoting the expression of proinflammatory factors, exacerbating inflammatory responses, and inducing severe pulmonary damage. This study first investigates the role of TLR4 in IAV+SA co-stimulated inflammatory injury of pulmonary epithelial cells, followed by a further exploration of TAK-242 (a TLR4 inhibitor) and TLR4 silencing on NF-κB signaling regulation, aiming to elucidate the molecular mechanisms underlying IAV+SA co-infection-induced pulmonary epithelial inflammatory injury.

## 2. Materials and Methods

### 2.1. Influenza A Virus

The virus strain used was the mouse lung-adapted strain of influenza A virus (A/PR/8/34) [25]. In the experiment, all experimental groups infected with influenza virus used the viral solution with a titer of 1:640, which was mixed with virus maintenance medium at a volume ratio of 1:100.

### 2.2. Bacterium Strain

The standard strain of *S. aureus* (SA) (ATCC 6539) was purchased from the Beijing Microbiological Cultural Collection Center (Beijing, China) and stored at −80 °C in the Pathogenic Microbiology Laboratory of Hunan University of Chinese Medicine (Changsha, China). For experiments, frozen stock tubes of SA were removed from the −80 °C freezer, thawed at room temperature, and streaked onto standard LB agar plates using an inoculation loop via quadrant streaking. The plates were cultured overnight at 37 °C, then stored short-term at 4 °C for subsequent use.

### 2.3. Cell Strain

The mouse lung epithelial cell line MLE-12 (Beijing Beina Chuanglian Biotechnology Co., Ltd., Beijing, China) was cultured in RPMI 1640 medium (RPMI 1640; Gibco, Waltham, WA, USA) supplemented with 10% fetal bovine serum (FBS; Wuhan Pricella Life Technology Co., Ltd., Wuhan, China). After passaging, the cells were used for subsequent experiments.

### 2.4. Detection of Influenza Virus Nucleoprotein mRNA Expression Levels and Post-IAV-Stimulated Lung Epithelial Cell Viability

MLE-12 cells were cultured in 10% FBS RPMI 1640 and added to 6-well or 96-well plates. The experiment included a negative control (NC) group and an IAV group. In the IAV group, cells were infected with IAV for 4 h, then replaced with cell maintenance culture medium (2% FBS RPMI 1640) and cultured for 24 h, 36 h, or 48 h. At these time points, cells from the 6-well plates were collected for the RT-qPCR detection of influenza virus nucleoprotein (NP) mRNA. RNA was extracted using the SteadyPure Quick RNA Extraction Kit (AG21013, Changsha, China), reverse-transcribed into cDNA with the NovoStart^®^ cDNA SuperMix Plus (E047-01B, Suzhou, China), and amplified using the NovoStart^®^ SYBR qPCR SuperMix Plus (E099-01A, Suzhou, China). The reagent concentrations and cycling conditions are shown in Tables S1 and S2. The reactions were performed on a Roche LightCycler^®^ 96 instrument (Hoffmann-La Roche AG, Basel, Switzerland). After completion, the melting curves were analyzed. GAPDH served as the internal reference gene, with relative quantification conducted through the 2^−ΔΔCT^ method. Cells in 96-well plates were used for cell viability assays using a CCK8 kit (Ecotop Biotechnology Co., Guangzhou, China) according to the manufacturer’s protocol, and absorbance at 450 nm was measured using a microplate reader. The primers are shown in Table S3.

### 2.5. Detection of Lung Epithelial Cell Viability and Levels of Cell-Free DNA and Citrullinated histone H3 After Co-Stimulation with IAV and SA

MLE-12 cells were cultured 10% FBS RPMI 1640 and added to 96-well cell plates. The experimental groups included NC (negative control), IAV, SA, and IAV+SA. The IAV group and IAV+SA group were infected with 1:100 diluted influenza virus suspension for 4 h, while the NC group and SA group received a virus maintenance medium during this period. All groups were then replaced with 2% FBS RPMI 1640 and cultured for 24 h or 36 h. The SA group and IAV+SA group were infected with SA suspension (MOI = 10) for 1 h, 2 h, or 3 h, while the NC group and IAV group received 2% FBS RPMI 1640. Cells and supernatants were collected for subsequent assays.

The cell viability assay was performed as described in Section 2.4 (consistent with the CCK8 method).

Cell-free DNA (cfDNA) Detection: Supernatants were centrifuged at 3000× *g* for 5 min. A total of 180 μL of supernatant was added to each well of a 96-well plate, followed by 20 μL SYTOX^®^ Green Nucleic Acid Stain (0.3 μM, Thermo Fisher Scientific, Waltham, WA, USA). Samples were incubated in the dark at room temperature for 15 min according to the manufacturer’s protocol, and the fluorescence intensity was measured using an MD fluorescence microplate reader (excitation: 504 nm; emission: 523 nm).

Citrullinated histone H3 (CitH3) Detection: CitH3 levels were quantified using a CitH3 ELISA kit (Shanghai Enzyme-Linked Biotechnology Co., Ltd., Shanghai, China) following the manufacturer’s instructions, and the absorbance was measured at 450 nm using a microplate reader.

### 2.6. Detection of mRNA Expression Levels of TLR4, NF-κB, and Inflammatory Cytokines in Lung Epithelial Cells Following Co-Stimulation with Influenza Virus and SA

MLE-12 cells were cultured in 10% FBS RPMI 1640 and added to 6-well plates. The experimental groups included the NC group, IAV group, SA group, and IAV+SA group. The IAV group and IAV+SA group were infected with 1:100 diluted influenza virus suspension for 4 h, while the NC group and SA group received a virus maintenance medium. After incubation, all groups were replaced with 2% FBS RPMI 1640 for 24 h. The SA group and IAV+SA group were then infected with SA suspension (MOI = 10) for 1 h/2 h/3 h, whereas the NC and IAV groups received 2% FBS RPMI 1640. After infection (1 h/2 h/3 h), cells were collected for RNA extraction and analyzed by RT-qPCR. The reagent kit, cDNA reaction system, cDNA amplification reaction steps, and calculation method were the same as in Section 2.4. The primers are shown in Table S3.

### 2.7. Detection of TLR4, NF-κB, and Inflammatory Cytokine mRNA Expression Levels in Pulmonary Epithelial Cells Following TAK-242 Treatment

MLE-12 cells were cultured in 10% FBS RPMI 1640 and added to 6-well plates. The experimental groups included control groups (NC, IAV, SA, and IAV+SA) and TAK-242 intervention groups (TAK-242-NC, TAK-242-IAV, TAK-242-SA, and TAK-242-IAV+SA). For the control groups, the IAV and IAV+SA groups were infected with 1:100 diluted influenza virus suspension for 4 h, while the NC and SA groups received a virus maintenance medium. All groups were then replaced with 2% FBS RPMI 1640 for 24 h. The SA and IAV+SA groups were subsequently infected with SA suspension (MOI = 10) for 3 h, while the NC and IAV groups received 2% FBS RPMI 1640. For the TAK-242 intervention groups, cells were pre-incubated with 100 nM TAK-242 (MedChemExpress, Monmouth Junction, NJ, USA) for 4 h, followed by the same infections as corresponding control groups. After 3 h of final intervention, cells were collected for RNA extraction and analyzed by RT-qPCR. The use of reagent kits, cDNA reaction system, cDNA amplification reaction steps, and calculation method was the same as in Section 2.4. The primers are shown in Table S3.

### 2.8. Detection of TLR4 (mRNA/Protein), NF-κB, and Inflammatory Cytokine mRNA in Pulmonary Epithelial Cells Post-TLR4 Silencing

MLE-12 cells were cultured in 10% FBS RPMI 1640 and added to 6-well plates. The experimental groups included control groups (NC, IAV, SA, and IAV+SA) and siRNA transfection groups (siRNA-NC, siRNA-IAV, siRNA-SA, and siRNA-IAV+SA). The siRNA sequences used in this study are shown in Table S4. For the control groups, infections were performed according to the methods described in Section 2.7. In the siRNA transfection groups, cells were transfected with 20 nM siRNA (Sangon Biotech Co., Ltd., Shanghai, China) using RNA TransMate (E607402; Sangon Biotech Co., Ltd., Shanghai, China) following the protocol provided in the manual, followed by 6 h of transfection. The siRNA-NC group was simultaneously treated with a negative control vector followed by the corresponding control group protocols. After 3 h of final intervention, cells were collected for RNA extraction and analyzed by RT-qPCR. The reagent kit, cDNA reaction system, cDNA amplification reaction steps, and calculation method were the same as in Section 2.4. The primer sequences are shown in Table S3.

Cellular proteins were prepared for Western blotting analysis of TLR4 protein expression. The proteins were electrophoresed using the Bio-Rad Mini-Protean PowerPac (Bio-Rad Laboratories, Hercules, CA, USA), transferred onto membranes, and blocked. Membranes were incubated with the primary antibody (1:1000; TLR4, Proteintech, 66350-1-lg, Wuhan, China) at 4 °C overnight. After washing and incubation with the secondary antibody, bands were visualized with the Amersham Imager600(General Electric Company, Boston, MA, USA), and relative protein expression levels were calculated using ImageJ software (version 1.48).

### 2.9. Statistical Analysis

Statistical processing were conducted with SPSS 26.0 followed by graphical visualization through GraphPad Prism 8.0, with all data presented as mean ± standard deviation (x ± s). For normally distributed data, intergroup comparisons were analyzed using one-way ANOVA, with post hoc pairwise comparisons using the least significant difference (LSD) test (for homogeneous variances) or the Games–Howell test (for heterogeneous variances). Non-normally distributed data were analyzed using non-parametric tests. Statistical significance was set at *p* < 0.05.

## 3. Results

### 3.1. Expression Level of Influenza Virus NP mRNA and Viability Changes in Pulmonary Epithelial Cells After Influenza Virus Stimulation

**Figure 1:**
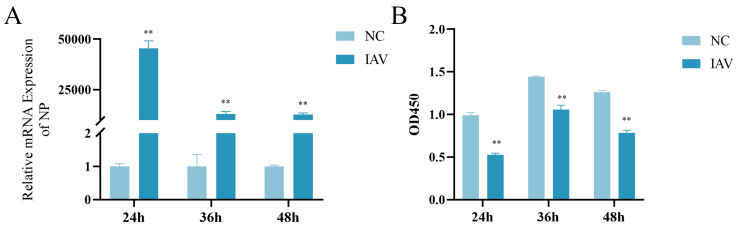
Effects of influenza virus stimulation on NP mRNA expression and viability changes in pulmonary epithelial cells. MLE-12 cells were cultured in 10% FBS RPMI 1640. After influenza virus infection, NP mRNA expression was assayed by RT-qPCR, and cell viability was measured using CCK-8 assay. (**A**) NP mRNA expression levels (*n* = 3). (**B**) OD values reflecting cell viability after influenza virus infection (*n* = 3). Compared with the NC group: ** *p* < 0.01.

RT-qPCR results showed that, compared with the NC group, the mRNA expression level of NP in the IAV group significantly increased at 24 h, 36 h, and 48 h after influenza virus stimulation in pulmonary epithelial cells. Notably, NP expression peaked at 24 h post-infection, followed by a gradual decline at 36 h and 48 h (Figure 1A). These findings suggest active replication of the influenza virus in pulmonary epithelial cells. Moreover, cell viability assays revealed that, compared with the NC group, the IAV group exhibited significantly decreased cell viability at 24 h, 36 h, and 48 h after viral stimulation (Figure 1B), indicating that influenza virus infection causes damage to pulmonary epithelial cells.

### 3.2. Effects of Influenza Virus and SA Co-Stimulation on Pulmonary Epithelial Cell Viability and Levels of Cell-Free DNA and Citrullinated histone H3

**Figure 2:**
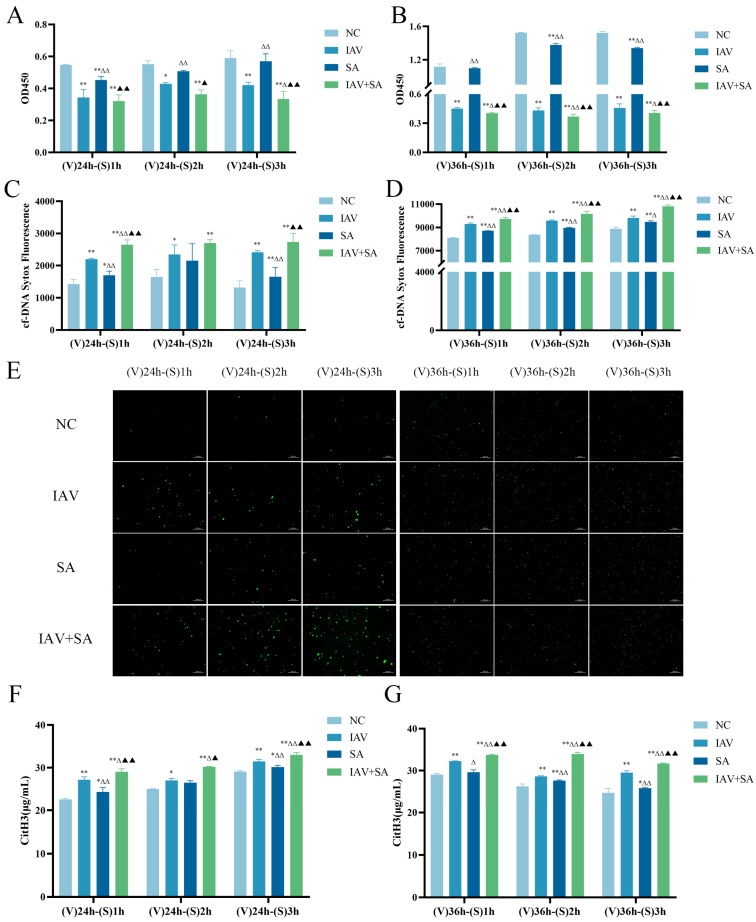
Effects of influenza virus combined with SA stimulation on lung epithelial cell viability and the levels of cfDNA and CitH3. MLE-12 cells were cultured in 10% FBS RPMI 1640. The IAV and IAV+SA groups were first infected with influenza virus for 4 h, followed by 2% FBS RPMI 1640 for 24 h/36 h. Subsequently, the SA and IAV+SA groups were stimulated with *S. aureus* for 1 h/2 h/3 h, with the NC group as a parallel control. Cell viability was measured by CCK-8 assay, cfDNA release was detected using SYTOX^®^ Green Nucleic Acid Stain (Thermo Fisher Scientific, Waltham, WA, USA), and CitH3 levels were quantified via ELISA. (**A**,**B**) OD values of cell viability (*n* = 3). (**C**–**E**) Detection results and fluorescence micrographs of cfDNA in cell supernatants (*n* = 3). (**F**,**G**) CitH3 levels (μg/mL) in cell supernatants (*n* = 3). Compared with the NC group: * *p* < 0.05, ** *p* < 0.01; compared with the IAV group: ^Δ^ *p* < 0.05, ^ΔΔ^ *p* < 0.01; compared with the SA group: ^▲^ *p* < 0.05, ^▲▲^ *p* < 0.01. (V): influenza virus stimulation; (S): SA stimulation.

Based on preliminary findings, we selected 24 h and 36 h post-IAV stimulation for subsequent SA exposure (1 h/2 h/3 h) to investigate their combined effects. Cell viability analysis revealed significantly reduced viability in the IAV+SA co-stimulation group compared with the other groups (Figure 2A,B), demonstrating that the combined viral–bacterial challenge exacerbates cellular damage.

To investigate the biological mechanisms by which influenza virus combined with SA stimulation affected cell viability, we measured the levels of cfDNA and CitH3. cfDNA is a hallmark indicator of cell death [26], whereas CitH3 is a critical marker of nuclear DNA disintegration during eukaryotic cell death [27]. SA, a prokaryotic organism, lacks histones in its nucleoid. Therefore, cfDNA and CitH3 levels reflect the extent of lung epithelial cell damage and cell death. The results showed that, compared with the NC group, all intervention groups exhibited significantly elevated cfDNA and CitH3 levels, with the IAV+SA group demonstrating the highest expression (Figure 2C–G). These findings indicate that both influenza virus and *S. aureus* alone induce cellular damage, whereas their combined stimulation exacerbates cell injury.

### 3.3. Effects of Influenza Virus Combined with SA Stimulation on TLR4 and Downstream Inflammatory Cytokine mRNA Expression in Lung Epithelial Cells

**Figure 3:**
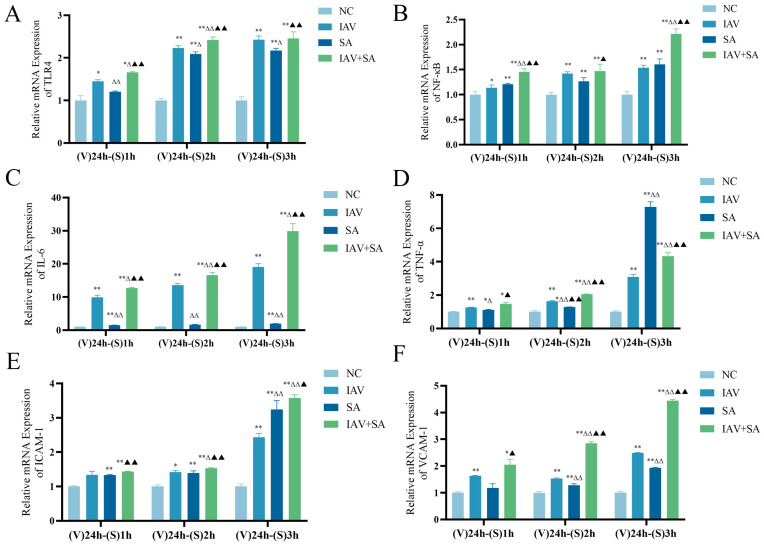
Effects of influenza virus combined with SA stimulation on TLR4, NF-κB, and inflammatory cytokine mRNA expression in lung epithelial cells. MLE-12 cells were cultured in 10% FBS RPMI 1640. The IAV and IAV+SA groups were first infected with influenza virus for 4 h, followed by 2% FBS RPMI 1640 for 24 h. Subsequently, the SA and IAV+SA groups were stimulated with SA for 1 h/2 h/3 h, with the NC group as a parallel control. mRNA expression levels of TLR4, NF-κB, and inflammatory cytokines were measured by RT-qPCR. (**A**–**F**) mRNA expression of TLR4, NF-κB, IL-6, TNF-α, ICAM-1, and VCAM-1 in cells (*n* = 3). Compared with the NC group: * *p* < 0.05, ** *p* < 0.01; compared with the IAV group: ^Δ^ *p* < 0.05, ^ΔΔ^ *p* < 0.01; compared with the SA group: ^▲^ *p* < 0.05, ^▲▲^ *p* < 0.01. (V): influenza virus stimulation; (S): SA stimulation.

Previous results have confirmed that combined influenza virus and SA stimulation caused severe cellular damage and death in lung epithelial cells. Both 24 h and 36 h influenza virus interventions followed by *S. aureus* stimulation yielded similar outcomes. Considering cell viability and experimental feasibility, we adopted a protocol of 24 h IAV intervention combined with 1 h/2 h/3 h *S. aureus* stimulation to assess inflammatory markers. The results demonstrated that, compared with the NC group, mRNA expression levels of TLR4, NF-κB, IL-6, TNF-α, ICAM-1, and VCAM-1 were elevated in the IAV, SA, and IAV+SA groups (Figure 3A–F). Except for TNF-α levels in the SA group at the (V) 24 h–(S) 3 h time point, which were higher than those in the IAV+SA group (Figure 3D), all other markers showed significantly higher mRNA expression levels in the IAV+SA group at all time points. Thus, compared with individual influenza virus or *S. aureus* stimulation, combined stimulation markedly activated TLR4 and downstream inflammatory factor expression.

### 3.4. Changes in mRNA Expression Levels of TLR4, NF-κB, and Inflammatory Cytokines in Pulmonary Epithelial Cells Following TAK-242 Treatment

**Figure 4:**
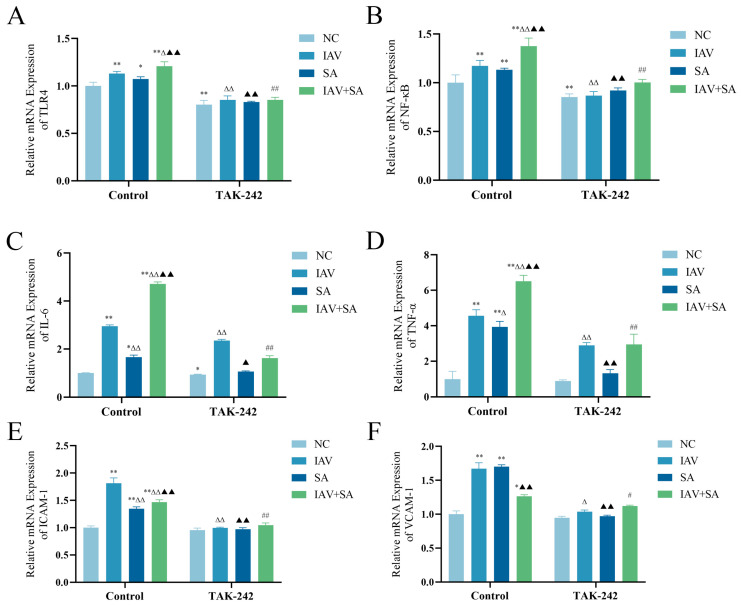
Changes in TLR4, NF-κB, and inflammatory cytokine mRNA expression levels in pulmonary epithelial cells following TAK-242 treatment. TAK-242 intervention groups (TAK-242-NC, TAK-242-IAV, TAK-242-SA, TAK-242-IAV+SA) were pretreated with TAK-242 for 4 h, followed by corresponding experimental protocols as in control groups. Cells were harvested 3 h post-intervention. mRNA expression levels were measured by RT-qPCR. (**A**–**F**) mRNA expression levels of TLR4, NF-κB, IL-6, TNF-α, ICAM-1, and VCAM-1 with or without 100 nM TAK-242 treatment (*n* = 3). Compared with the NC group: * *p* < 0.05, ** *p* < 0.01; compared with the IAV group: ^Δ^ *p* < 0.05, ^ΔΔ^ *p* < 0.01; compared with the SA group: ^▲^ *p* < 0.05, ^▲▲^ *p* < 0.01; compared with the IAV+SA group: ^#^ *p* < 0.05, ^##^ *p* < 0.01.

Previous findings have indicated that the co-stimulation of pulmonary epithelial cells with influenza virus and SA activates TLR4 and modulates the expression of downstream inflammatory factors. As TAK-242 is a specific inhibitor of TLR4 [28,29], we pretreated cells with TAK-242 prior to viral–bacterial co-stimulation to verify whether TLR4 regulates these inflammatory markers. The results demonstrated that TAK-242 intervention significantly reduced TLR4 mRNA levels compared with those in the corresponding control groups without the inhibitor (Figure 4A). Furthermore, mRNA expression levels of the downstream TLR4-associated markers NF-κB, IL-6, TNF-α, ICAM-1, and VCAM-1 were downregulated in the TAK-242-treated group (Figure 4B–F). These results suggest that TAK-242 suppresses TLR4 activation and downstream signaling, confirming TLR4’s regulatory role in these inflammatory pathways.

### 3.5. Changes in TLR4, NF-κB, and Inflammatory Cytokine mRNA Expression Levels and TLR4 Protein Expression in Pulmonary Epithelial Cells Following TLR4 Gene Silencing

**Figure 5:**
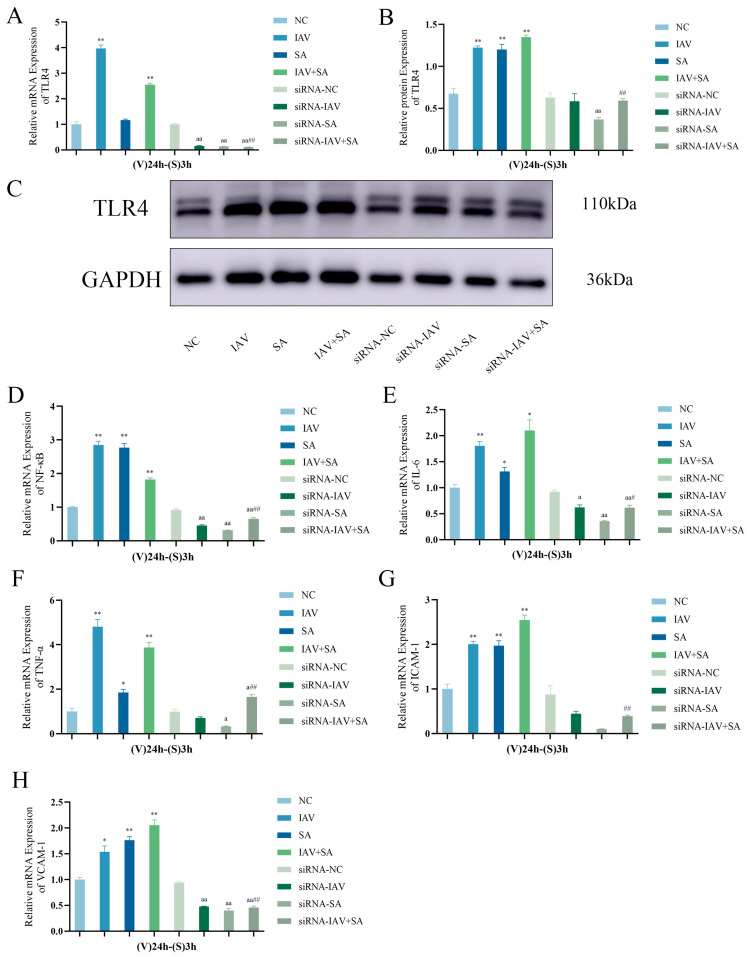
Changes in TLR4, NF-κB, inflammatory cytokine mRNA expression levels, and TLR4 protein expression levels in lung epithelial cells after TLR4 gene silencing. siRNA transfection groups (siRNA-NC group, siRNA-IAV group, siRNA-SA group, siRNA-IAV+SA group) were treated with TLR4-silencing siRNA for 6 h, while the siRNA-NC group was simultaneously treated with a negative control vector. Subsequent interventions followed the respective experimental protocols. Cells were collected 3 h post-intervention. RT-qPCR and Western blotting were used to verify TLR4 silencing efficiency, and RT-qPCR assessed mRNA expression changes in downstream markers. (**A**) TLR4 mRNA levels across groups after siRNA treatment (*n* = 3). (**B**,**C**) TLR4 protein expression levels across groups after siRNA treatment (*n* = 3). (**D**–**H**) NF-κB, IL-6, TNF-α, ICAM-1, and VCAM-1 mRNA expression levels post-siRNA treatment (*n* = 3). Compared with the NC group: * *p* < 0.05, ** *p* < 0.01; compared with the siRNA-NC group: ^a^ *p* < 0.05, ^aa^ *p* < 0.01; compared with the IAV+SA group: ^#^ *p* < 0.05, ^##^ *p* < 0.01. (V): influenza virus stimulation; (S): SA stimulation.

Previous studies demonstrated that TAK-242 treatment suppresses influenza virus-and SA-induced inflammatory cytokine mRNA expression in pulmonary epithelial cells. To further validate this mechanism, TLR4 was silenced in these cells using siRNA, followed by the analysis of TLR4 mRNA/protein levels and downstream inflammatory markers. RT-qPCR and Western blotting confirmed that siRNA transfection significantly inhibited TLR4 expression (Figure 5A–C). Subsequent evaluation of NF-κB, IL-6, TNF-α, ICAM-1, and VCAM-1 mRNA levels revealed that TLR4-silenced cells exhibited a markedly reduced expression of these markers upon viral–bacterial co-stimulation (Figure 5D–H). These findings further corroborate TLR4’s regulatory role in pulmonary epithelial inflammatory responses triggered by influenza virus and *S. aureus* co-infection.

## 4. Discussion

This study investigated the effects of co-stimulation with IAV and SA on lung epithelial cells using in vitro experiments. The results revealed that influenza virus replication caused damage to lung epithelial cells. Dynamic changes in the NP suggest that the virus reaches its replication peak during the early stages of infection. IAV RNA synthesis appeared to be most active at 24 h post-infection, whereas the viral replication rate decreased at 36 h and 48 h, likely due to factors such as cell death and the accumulation of viral metabolites.

Assays measuring lung epithelial cell viability, cfDNA, and CitH3 levels after 24 h/36 h of IAV exposure, followed by SA co-stimulation, revealed that the IAV+SA group exhibited the most severe cellular damage, followed by the IAV and SA groups. This may be attributed to the increased pathogen load and prolonged stimulation duration in the IAV+SA group, as well as the potential synergistic pathogenic effects between the influenza virus and SA. And cfDNA and CitH3 can serve as biomarkers of cellular infection, inflammatory status, and pathological injury [30,31]. This study demonstrated that the IAV+SA group exhibited the highest levels of cfDNA and CitH3, indicating severe cellular damage, cell death, and enhanced release of free DNA and CitH3.

TLR4, a critical regulator of inflammatory responses, plays essential roles in viral and bacterial infections [32,33,34,35]. NF-κB, a ubiquitously expressed nuclear transcription factor and downstream effector of TLR4, promotes the release of inflammatory cytokines and mediates diverse inflammatory cascades [35]. Inflammatory cytokines such as TNF-α and IL-6, released post-influenza infection, can further enhance bacterial adhesion and invasion [36]. These cytokines are key contributors to disease severity. In this study, we observed significantly elevated expression levels of TLR4, NF-κB, IL-6, and other mediators in the IAV+SA group. We hypothesize that co-stimulation of lung epithelial cells with influenza virus and SA activates TLR4, triggering the expression of inflammatory mediators like NF-κB, IL-6, TNF-α, ICAM-1, and VCAM-1. Excessive upregulation of these mediators may drive hyperactive inflammatory responses leading to cellular damage and death. Consistent with previous research findings, the expression of TLR4 significantly increases after viral or bacterial infection, and the expression of its downstream related indicators also markedly rises, which can lead to more severe pathological damage. This demonstrates that influenza virus may promotes *S.aureus* infection in lung epithelial cells and exacerbates pulmonary inflammatory responses. The present study further confirms the critical role of TLR4 in viral and bacterial infections.

Studies have demonstrated that the downregulation of TLR4 reduces the expression of inflammatory factors such as IL-6 and ICAM-1 [37]. TAK-242 can inhibit lipopolysaccharide-induced endothelial inflammatory responses by downregulating IL-6, ICAM-1, and VCAM-1 expression [38,39], while NF-κB participates in regulating ICAM-1 and VCAM-1 gene expression [40,41]. Previous studies have shown that knocking out TLR4 can inhibit inflammatory responses to alleviate acute liver injury [42]. This study employed the TLR4 inhibitor TAK-242 and TLR4 gene silencing via siRNA to investigate their effects on IAV and SA co-stimulated lung epithelial cells. The results indicated that both TLR4 inhibition and gene silencing significantly decreased TLR4 expression. The TAK-242/siRNA-treated IAV+SA group exhibited markedly decreased mRNA levels of inflammatory factors (NF-κB, IL-6, TNF-α) and adhesion molecules (ICAM-1, VCAM-1) compared with the uninfected IAV+SA group, confirming TLR4 as the key receptor mediating co-infection-induced inflammation. Suppressed TLR4 expression downregulated NF-κB, verifying TLR4 as an upstream regulator of NF-κB. This inhibition cascade subsequently reduces the levels of inflammatory factors and adhesion molecules, blocks pathogen-induced excessive immune activation, and alleviates cellular pathological damage. Consistent with previous research findings, the use of TLR4 inhibitor TAK-242 or siRNA-mediated TLR4 silencing can suppress TLR4 activation, leading to a downregulation of its downstream indicators and thereby alleviating inflammatory responses.

In summary, this study proposes that IAV+SA stimulation in lung epithelial cells induces a more pronounced inflammatory response compared with IAV or SA stimulation alone. IAV+SA primarily promotes TLR4 expression, activates the TLR4/NF-κB signaling pathway, and upregulates the secretion of proinflammatory factors, thereby exacerbating lung epithelial cell damage. However, upon using the TLR4 inhibitor TAK-242 or silencing TLR4 with siRNA, NF-κB activation is subsequently inhibited, leading to reduced expression levels of downstream inflammatory cytokines and adhesion molecules associated with the TLR4/NF-κB signaling pathway. This demonstrates potent anti-inflammatory effects, which are beneficial for mitigating the intensity of inflammatory responses induced by the combined infection of influenza virus and *S.aureus*, as well as alleviating cellular inflammatory reactions.

## 5. Conclusions

This study demonstrated that co-stimulation with IAV and SA activates TLR4, upregulates inflammatory factor expression in lung epithelial cells, and exacerbates inflammatory injury. However, this research did not fully elucidate the specific mechanisms of TLR4-mediated inflammatory regulation or its in vivo role in mice and was limited to an in vitro experimental model. Future studies should investigate the precise mechanisms by which TLR4 regulates inflammatory factors under dual stimulation by IAV and SA.

Currently, most studies on pneumonia focus on a single pathogen, whereas this research adopts a dual-pathogen co-intervention approach. Additionally, in vitro studies of bacterial pneumonia predominantly utilize LPS for modeling, with rare applications involving live bacteria. Moreover, most research on post-influenza secondary pneumonia has focused on in vivo models, with limited in vitro exploration. This study adopts an in vitro experimental approach, utilizing IAV and live *Staphylococcus aureus* to reveal the mechanisms of inflammatory injury caused by IAV+SA co-stimulation. This approach provides a novel reference for in vitro studies of influenza-associated secondary bacterial pneumonia and offers experimental insights into the prevention and treatment of post-influenza secondary pneumonia.

## Data Availability

The original contributions presented in this study are included in the article/Supplementary Material. Further inquiries can be directed to the corresponding author.

## Supplementary Materials

The following supporting information can be downloaded at https://www.mdpi.com/article/10.3390/microorganisms13061201/s1, Table S1. Composition of cDNA amplification reaction; Table S2. Step of cDNA amplification reaction; Table S3. Primers used in this study; Table S4. The siRNA sequences used in this study.

## Abbreviations

The following abbreviations are used in this manuscript:

ALI	Acute lung injury
ARDS	Acute respiratory distress syndrome
CitH3	Citrullinated histone H3
cfDNA	Cell-free DNA
ELISA	Enzyme-linked immunosorbent assay
FBS	Fetal bovine serum
IAV	Influenza A virus
ICAM-1	Intercellular adhesion molecule 1
IL-6	Interleukin 6
LPS	Lipopolysaccharide
LSD	Least significant difference
MOI	Multiplicity of infection
NC	Negative control
NF-κB	Nuclear factor kappa-light-chain-enhancer of activated B cells
RT-qPCR	Reverse transcription quantitative polymerase chain reaction
SA	*Staphylococcus aureus*
siRNA	Small interfering RNA
SPSS	Statistical Package for the Social Sciences
TAK-242	Resatorvid
TLR4	Toll-like receptor 4
TNF-α	Tumor necrosis factor alpha
VCAM-1	Vascular cell adhesion molecule 1
