# Supplementary material for: The Role of TLR4 in Lung Epithelial Cell Injury Caused by Influenza Virus Combined with Staphylococcus aureus
Source: Microorganisms. 2025 May 24;13(6):1201. doi: 10.3390/microorganisms13061201 (PMC12195505; doi:10.3390/microorganisms13061201)
Supplement: Supplementary file 1 [file microorganisms-13-01201-s001.zip › microorganisms-3616635-supplementary.pdf]

Supplementary Information

The Role of TLR4 in Lung Epithelial Cell Injury Caused by Influenza Virus Combined with *Staphylococcus aureus*

Bei Chen [1], Chunjing Chen [1], Fangguo Lu [1,2,*], Xiaoqi Wang [2], Xianggang Zhang [2], Zhibin Wang [2] and Huihui Liu [1]

[1] Medical School, Hunan University of Chinese Medicine, Changsha 410208, China; 20223769@stu.hnucm.edu.cn (B.C.); 004787@hnucm.edu.cn (C.C.); liuhuihui916@163.com (H.L.)
[2] School of Integrated Chinese and Western Medicine, Hunan University of Chinese Medicine,
Changsha 410208, China; 20232123@stu.hnucm.edu.cn (X.W.); 20222126@stu.hnucm.edu.cn (X.Z.); 15680801830@163.com (Z.W.)
* Correspondence: 001196@hnucm.edu.cn; Tel.: +86-13647310305

Table S1. Composition of cDNA amplification reaction

Reagents	Volume
2×NovoStart® SYBR qPCR SuperMix Plus	10 μL
Forward primer (10 mM)	2 μL
Reverse primer (10 mM)	2 μL
cDNA	1 μL
RNase Free Water	5 μL

Table S2. Step of cDNA amplification reaction

Steps	PCR reaction conditons
Pre-denaturation	95℃,1min
Denaturation,annealing,extensio	95℃ 20s→60℃ 20s→72℃ 30s,40-45cycles
Melting curve	95℃ 10s,65℃ 60s,97℃ 1s

Table S3.Primers used in this study.

Target Gene	Orientations	Primer Sequence(5′-3′)
NP	F	CCTGTGTGTATGGACCTGCC
	R	CTCTTGGGACCACCTTCGTC
TLR4	F	CCGCTTTCACCTCTGCCTTCAC
	R	ACCACAATAACCTTCCGGCTCTTG
NF-κB	F	ATGGGAAACCGTATGAGCCTGTG
	R	AGTTGTAGCCTCGTGTCTTCTGTC
IL-6	F	GACTTCCATCCAGTTGCCTT
	R	ATGTGTAATTAAGCCTCCGACT
TNF-α	F	AGCACAGAAAGCATGATCCG
	R	CACCCCGAAGTTCAGTAGACA
ICAM-1	F	GCAAGAAGATAGCCAACCA
	R	TGCCAGTTCCACCCGTTC

VCAM-1	F	GAGGGTGGTGCTGTGACAATGAC
	R	GGGTGGCATTTCCTGAGAGAAGC
GAPDH	F	GGTTGTCTCCTGCGACTTCA
	R	TGGTCCAGGGTTTCTTACTCC

Note: All primers were designed and synthesized by Sangon Biotech Co., Ltd., Shanghai, China. (https://www.sangon.com/). The specific synthesis procedure can be verified on the official website.

Table S4.The siRNA sequences used in this study

Name	Species	Forward(5'-3')	Reverse(5'-3')
siRNA-TLR4	mouse	GGAUCUUUCUAAAUGUCAATT	UUGACAUUUAGAAAGAUCCTT
siRNA-NC	mouse	UUCUCCGAACGUGUCACGUTT	ACGUGACACGUUCGGAGAATT

Note: All the siRNA sequences were designed and synthesized by Shanghai Sangon Biotech Company (https://www.sangon.com/). The specific synthesis procedure can be verified on the official website.
